# The Evolving Context of MAID-Related Communications for Registered Nurses in Canada

**DOI:** 10.1177/08445621241281993

**Published:** 2024-09-09

**Authors:** Sally Thorne, Heidi Parker, Barbara Pesut

**Affiliations:** 1 70439 School of Nursing, University of British Columbia, Vancouver, BC, Canada; 2 104259 School of Nursing, University of British Columbia, Okanagan, Kelowna, BC, Canada

**Keywords:** Assisted dying, end-of-life, death & dying, communication, person centered care

## Abstract

**Background:**

Since legalization of Medical Assistance in Dying in Canada in 2016, nurses are increasingly faced with new and evolving communication challenges as patients in a diversity of settings and contexts contemplate their end-of-life options.

**Purpose:**

The purposes of this study were: 1) to develop an understanding of the nuances and challenges associated with MAID-related communication from the perspective of registered nurses, and 2) to draw on the insights arising from this analysis to reflect on the evolution of MAID communication for nurses over time.

**Methods:**

This study represented a secondary analysis of two primary qualitative data sets, including: 74 interviews of Canadian registered nurses self-identifying as having some exposure to MAID in their clinical practice; and 47 narrative reflections volunteered by respondents to questions posed in an online MAID reflective guide for nurses.

**Results:**

Nurses described evolving complexities associated with introducing and engaging with the topic of MAID with their patients, helping patients navigate access to MAID assessment, managing family and community dynamics associated with opinions and beliefs surrounding MAID, supporting patients in their planning toward a MAID death, and being there for patients and their families in the moment of MAID.

**Conclusions:**

MAID communication is highly complex, individualized, and context-specific. It is apparent that many nurses have developed an impressive degree of comfort and skill around navigating its nuances within a rapidly evolving legislative context. It is also apparent that dedicated basic and continuing MAID communication education will warranted for registered nurses in all health care settings.

## Background & purpose

### Introduction to the Canadian MAID context

When changes to Canada's Criminal Code made Medical Assistance in Dying [MAID] a legal option in June of 2016, the health care system was somewhat taken by surprise (Winters et al., 2021). Although the issue had long been discussed and debated in the public domain and in court challenges, most Canadians anticipated that the journey toward creating a legal framework would be long and tortuous. Since the context of this change in legislation included a ruling the year prior that the current Criminal Code must satisfy the Canadian Charter of Rights and Freedoms, the change not only signaled a legally permissible medical action, but also a procedure for which Canadians who met the eligibility requirements could expect access (Dumsday, 2023). In the scramble to adapt to these new conditions, the provincial and territorial Ministries of Health, who hold responsibility for funding and governing health care systems, the regional health authorities within provinces and territories, and the provincial and territorial health professional regulatory bodies all took up the challenge. The initial legislation (Government of Canada, 2016) has evolved over these years, primarily to expand eligibility beyond those for whom natural death is reasonably foreseeable to now include those who have a grievous and irremediable medical condition which is in irreversible decline, reasonable treatments to relieve suffering have been tried and failed, and for whom suffering cannot be relieved in a manner that is reasonable to the patient (Government of Canada, 2021). However, the challenge across the system has been to create the conditions under which those who are eligible and seek MAID can have access, and the care of all patients who may be on a trajectory toward considering MAID is fully supported (Downar et al., 2020; Wiebe et al., 2021).

Assessments for eligibility and provision of MAID in Canada can only be performed by physicians [MDs] or nurse practitioners [NPs] (Health Canada, 2023a). In the Canadian context, NPs have specialized advanced clinical education at the graduate degree or certificate level and are governed by a distinct regulatory framework with authority to autonomously diagnose and treat illnesses and perform various medical procedures across the lifespan (Acorn & Byres, 2020). Although many do work in interprofessional teams, the NP licensure affords them the right to work independently without a supervising physician. While Canadian legislation allows for two forms of MAID – that which is provided by an MD or NP and that which is self-administered based on prescription – it has become apparent over these initial years that the vast majority of MAID is enacted through provision by a qualified health care professional (Health Canada, 2023b).

### Communication as a critical component of MAID care

All health care professionals involved in MAID care have had to learn new communication skills as part of their engagement with these patients (Wiebe et al., 2021). In some respects, that communication is tightly controlled by legislative conditions, such as requiring careful attention to the Criminal Code prohibition against counselling a person to die by suicide (Government of Canada, 2024), which remains an indictable offense with certain explicit exemptions provided to that law for those with specific legislated roles in the case of MAID (Health Canada, 2024b). At the same time, health care professionals have an explicit responsibility to “respond to patients’ questions and requests for information about MAID either directly themselves or by ensuring contact with an individual or a MAID program which can provide the information” (Health Canada, 2023a, section 14, para 2). Although the legislation makes provision for health care professionals to abstain from being involved in MAID provision, they are prohibited from engaging in such a manner as to create barriers to MAID access.

While the majority of formal guidance and regulatory practice standards for health care professionals are concerned with those with the authority for MAID eligibility assessment and provision, and an accredited national curriculum is available for NPs and MDs (Canadian Association of MAID Assessors & Providers [CAMAP], 2022), there are many other health care professionals who encounter patients across a wide range of settings who may be considering or requesting access to MAID. These individuals have had to adapt to this new form of practice, including trying to understand the implications of the evolving legislation and practice standards of their NP and MD colleagues, generally with relatively few supports and resources (Banner et al., 2019; Pesut et al., 2019, 2020b). Although provincial nursing practice standards are beginning to become more robust and evidence based, many registered nurses [RNs] have had to figure out the boundaries, constraints, and challenges of their role in this respect through trial and error (Parker, 2024; Pesut et al., 2020c)**.** Patients who are or may become eligible for MAID encounter RNs across many different contexts (including acute care, community or home care settings or residential longterm care settings). For many of these patients, RNs may represent the most intimate and continuing of health care professional services. Therefore, large numbers of RNs who did not foresee MAID as playing a role in their practice are having to confront its implications for the care they offer (Beuthin et al., 2018; Hébert & Asri, 2022).

### Purpose

The main purpose of this secondary qualitative analysis (Thorne, 2013) of data from two distinct data sets was to begin to understand some of the nuances and challenges associated with MAID-related communication from the perspective of RNs who are not MAID specialists in the sense of not being directly involved in MAID assessment and provision or, in most cases, having any formal training in this practice. As a secondary aim, we sought to draw on the insights arising from this analysis to reflect on the evolution of MAID communication for these RNs as the practice and legislative situation have evolved over time since MAID was first legalized in Canada. Therefore, the research question driving this secondary analysis study was as follows: What can be learned from the experiential accounts of RNs about the evolving complexities of communication in the context of MAID?

## Methods and procedures

### Data sources

For this secondary analysis we drew on two data sets that had been developed in the course of an ongoing program of inquiry into nursing perspectives on the implications of MAID for their practice with patients. The first was a set of over 120 interviews with nurses (including both RNs and NPs) across Canada conducted 2017 through 2023 from which various findings not specific to patient communication have been previously published (Pesut et al., 2020c, 2020d, 2021, 2024). The second data set arose from voluntary anonymous submissions to an online “MAID Reflective Guide for Nurses” we developed as a knowledge translation activity on the basis of the interview study data analysis (Pesut et al., 2023). As an option, those working through the reflective guide could select to have their written reflections made available to us for the purposes of further research. The guide was explicitly designed to be openly accessible without self-identification requirements, and while it was primarily targeted toward RNs, we became aware that some who found it useful then recommended it to colleagues in other health professions. Both studies received ethical approval from our institutional review board. The resultant body of material from which the current secondary analysis is drawn therefore includes: 1) transcripts of individual interviews (primarily phone or virtual) with 74 RNs, and 2) segments of narrative reflections from 47 individual anonymous respondents to the reflective guide (presumably all or most being RNs). Our interview sample was strategically diverse, in that it included RNs who identified as “conscientious objectors” with respect to MAID, those who were supportive of MAID as an option, and those who had not yet formed a clear opinion. It included RNs with extensive experience with MAID and those who had singular or occasional involvement. It included both RNs who were actively engaged with the MAID process (such as preparing for the event and starting the IV) and others whose involvement with the MAID aspect was more peripheral to their focus of care. In some instances, these RNs had longstanding and extensive involvement with such patients, and in others the interactions with the patient were occurring in a rapidly developing relational context specific to end-of-life care in general or MAID in particular.

### Data analysis

In keeping with the initial studies, we used Interpretive Description methodology (Thorne, 2016) to guide our analytic process. We engaged in constant comparative analysis as we inductively reflected on the corpus of available data, sorted and organized general thematic groupings within the data, and on this basis generated a schema for the presentation of these grouped findings that is consistent with distinct components of the MAID trajectory within which RNs encounter patients. Our emphasis in this analysis was on the evolving insights arising from the practice of these RNs as they increasingly engaged with patients in this context and as the legislative, regulatory and practice standards landscape evolved over time. Thus, although the data sets contained a wide range of perceptions, experiences and reflections from the perspective of these RNs, we extracted from that data set the material we interpreted as pertaining to the communication challenge, primarily in relation to RN-patient communication, but also extending to communications with families, the team, and one another.

## Results

What we learned from this secondary analysis highlighted the communication complexities, from an RN perspective, relative to the many different contexts in which RNs engage with patients around MAID. Our findings are therefore organized around these contextual dimensions or phases within the MAID trajectory so that the nature of the communication challenges and the impact of the evolving practice context can be illuminated and interpreted.

### Introducing the topic

When MAID legislation was first enacted, many RNs assumed that it was unacceptable to make any reference to the practice until the patient had first clearly initiated the topic. In some Canadian provinces, that assumption was reinforced in early practice standards, in some instances both not only for RNs, but also for NPs and MDs (Pesut et al., 2020d). However, in an era in which equity concerns are top of mind for the nursing profession, many RNs quickly realized that patients with confidence and social privilege were far more likely to raise the topic than were those made marginal from the dominant population by virtue of ethnicity, culture, or other social determinants of health. Therefore, the profession quickly recognized that equitable care would require some capacity to initiate such conversations in order to ensure that those who might be eligible for a care option to which they had a right also had the opportunity to exercise that right (Canadian Nurses Protective Society, 2021).

In this context, RNs described a complex process of trying to respond to cues from patients with respect to the nature of conversations they were seeking and the care options about which they might require information. “We’re always attending to what a person holds as goals, as values, as dear to the journey that they’ve never taken before and sometimes never considered before. And so, therefore may not have the words for.” As one explained, “It's taking the direction from the patient and creating space.” By virtue of the nature of their involvement with patients (such as those middle of the night occasions when patients asked when suffering might end), they often saw opportunities that may not have been discernable to other members of the health care team. As one RN expressed it, “You're noticing body language, you're noticing somebody tense up, you're noticing somebody look away, and that guides the ship of how I'm going to ask questions and if I'm going to ask questions.” Another explained, “There has to be something that leads you to that conversation.” In contrast, especially as challenges with equitable access became apparent, others felt strongly that it was a nursing responsibility to initiate the conversation in some circumstances. One RN recalled a case in which a patient who had been under her care for three weeks said “I’ve been wondering when this conversation would come up and when would you bring it up.” However, since counselling to suicide remains a prohibited act, they always proceeded with exceptional caution. For many, opening up general conversations with respect to goals of care, or end-of-life options (i.e., a palliative approach to care (Sawatzky et al., 2016)) became a way to enter these discussions, always ensuring that they took a lead from patient's beliefs and values, and proceeding cautiously into providing options for care at a pace that was appropriate to the context.

RNs also understood the importance of documenting and sharing the substance of these conversations with the health care team so that patients could be treated with a common and supportive approach to their care decisions. Because they also understood that suffering was a central criterion for MAID eligibility in the Canadian legislative context, they ensured that they engaged in conversations with patients to better understand the nature and form of their suffering, and documented that in a manner that would help substantiate the patient's situation should a request for MAID arise. Therefore, introducing the topic became a highly complex, delicate, and individualized process, comprised of judgements around who ought to be having which conversations with the patient, when, and with what level of detail. And in many instances, these RNs found themselves thrust into those introductory conversations whether they had planned to or not simply because their trusted relationships with patients led to their being the recipient of the patient's direct or indirect indication that such a conversation was needed.

### Navigating access

When patients indicated that information on or a conversation about MAID might be welcome, RNs were often in the position of having to help navigate access. Although RNs are not designated assessors or providers in the formal MAID context in Canada, many quickly realized that they had an obligation to their patients to become familiar with eligibility criteria so as not to unduly raise hopes or expectations in patients who might not be eligible. Therefore, many found themselves informally assessing the patient's situation against eligibility requirements in order to support patients toward accessing MAID services or helping them understand why that step might not be appropriate at this stage of their illness. This aspect of informal assessment became increasingly complex, and also important, when legislation permitting access for patients whose natural death was not reasonably foreseeable was enacted in 2021. As MAID is not yet available to persons whose suffering primarily derives from a mental health condition rather than a physical disease or disorder (Health Canada, 2024a), the complexities around helping patients with expectations have increased, and many RNs feel an increasing responsibility to provide active support for patients early on so that they understand what options may and may not be available to them. In addition, RNs began to appreciate the importance of being able to provide explanations about many of the other complex elements of the MAID process, such as interpreting mandatory waiting periods, or obtaining a waiver of final consent for provision should cognitive decline or pain management make the capacity for final consent in that moment uncertain. As one said, “We're able to be there and see their journey and help them through that journey. The ability to understand what their suffering is, is it's so important to be able to help them every single day through that.”

Another navigational aspect described by these RNs had to do with assisting patients and their families to overcome structural or attitudinal barriers to access. One recalled such an example, “They were going to send the forms in via post office and I was, like, ‘Oh, I think maybe, like, if you're okay with it, maybe I'll just fax it in so that it doesn't take, like, a week to get there.’ And so, I just kind of helped navigate them through that.” Since individual health professionals have the option to declare themselves to be conscientious objectors (Pesut et al., 2020a), some patients found themselves caught in relationships with health care professionals who discouraged conversations about end-of-life options or made it difficult to obtain information. Further, some institutions, especially some faith-based hospitals or hospices, were initially highly reluctant to allow access to information about MAID for patients in their facilities (Close et al., 2023). RNs in such settings therefore needed to know what publicly available information patients and their families might have access to, and in some instances, help directly broker that access. In the early days of MAID in Canada, some designated palliative care facilities did not permit access to MAID, feeling that the two practices were mutually exclusive (Downar et al., 2023). Over time, it seems apparent that MAID is becoming understood as highly consistent with an overall palliative care philosophy within which support is provided for patients to access all end-of-life services that may be consistent with their goals of care and legally available (Joolaee et al., 2022).

### Managing family & community dynamics

As health care professionals who work in palliative care have long recognized, family and community dynamics are often heightened in the intensity of an end-of-life process. Where MAID is (or may be) an option, these same kinds of dynamics can become extreme, particularly where different family members hold different beliefs as to the morality of assisted dying, or have differing convictions with respect to how processes ought to be managed. RNs are highly attuned to the complexities associated with some faith-based communities, particularly where there is categorical prohibition against hastening what is seen as the natural order of death. An RN recalled one such case, “He's interested and he's asking for more information and the wife or the family member is shutting him down, so therein lies that dilemma.”

Thus, for many RNs, an important component of supporting their patients along the journey toward MAID provision involves working closely with them, and sometimes with family members and other community members, to try to resolve what can be resolved, and protect the patient to the extent possible from undue distress and disturbance during this sensitive phase of life.

Because Canadian legislation places MAID decisions within the context of the autonomy of persons, there is no obligation for patients to inform their families of their decisions, and in such circumstances, health care professionals are governed by the usual rules around patient confidentiality (Wiebe et al., 2021). Although in most instances, patients and families try to work through these complexities together, some RNs do encounter the difficult situation of a patient who is proceeding with a plan for which the family will be kept in the dark. Therefore, in the spirit of trying to calm or resolve as much of the predictable family tension as possible, many do try to find ways to engage relationally with all of the key players, and to support as open a conversation as possible toward the ideal of a shared agreement on the process toward death. The priority for RNs is protecting their patient from the drama and intensity that can sometimes further compromise their emotional and psychological safety in the final weeks and days of living. Therefore, many find themselves in conversations with patients about who will be told, how they will be told, and how information will be managed to ensure as safe and smooth a progression as possible toward the desired end point. As one reflected, “It's, like, I am here to listen to your story, to partner with you in what you need, and be present with you.” Another explained, “It makes the patient's journey easier knowing that they have the love and support of their family, so that's how I introduce the topic.”

### Supporting planning toward the day

Among the MAID-related communications in which RNs engaged with their patients, a significant number had to do with planning toward the day of the MAID provision. While RNs may not always have been involved in the provision itself, they were often intimately engaged with patients and families in providing what they considered a complete ‘envelope of care’ so that the time between eligibility confirmation and the provision itself were as productive, meaningful and comfortable for all concerned. In many instances, this included discussions around what conversations patients might wish to have with their loved ones in advance of that date, messages they might wish to leave behind for after their death, and orchestrating the plan for the day itself in terms of who would be present, and for which aspects of the provision, how the physical environment where provision would take place might be made as warm and comfortable as possible. One described it as “Including all those human things we can bring to the moment. You know, would you like some soft music? Would you like your favourite blanket? Would you like to wear your forestry officer uniform? Would you like to have your family on your left or your right?” For some patients, this planning could involve a favorite last meal with family, specific music being played at the time of death, or other rituals and ceremonies to support both the patient and those left behind. RNs sometimes offered suggestions for considerations in this planning that the patient may not have thought about, or might have assumed were unavailable, but which became an important part of the plan. The patient's capacity to engage in orchestrating the death day, often with the creative support of the RNs caring for them in the days leading up to it, was a powerful signifier of the dignity and autonomy with which they had lived their life and intended to have in their death.

As with all things in health care, some plans do not quite unfold as intended or in the timing intended. The MD or NP scheduled for provision might become ill or caught up in other urgent care situations, or the date and time might require adjustment because of unanticipated conflicts. Adjusting to unplanned changes in circumstance and timing could be particularly sensitive for patients and families at this time, and RNs experienced considerable distress themselves if they could not make things flow as they should. Therefore, actively supporting patients and families through all of these complexities was a high priority in their interactions. At times, this might involve advocating for their patients should others in the system not quite recognize the significance of these modifications to a carefully orchestrated patient and family plan.

Another aspect of RN communication with respect to supporting patients and families toward the day involved preparing them for after care, such as a funeral home that had been pre-booked for transport, or certifying an expected death at home (a document that has come to replace the visit by a coroner that was requisite in the early years of MAID implementation). They often helped families understand how to access bereavement support options available in their institutions or communities. In the midst of all of this complexity, many RNs also found that they had a profound need to say their own good-bye to their patient.

### Being there in the moment

Although many NPs and MDs who do the MAID provision make it part of their practice to be with the patient and family for some time prior to and following the death, others do not, and may come and go in a relatively short time frame. RNs therefore often planned an approach to care that was based on a nuanced understanding of the individual patient and family needs and preferences for support, conversation, or privacy. As one explained, “Your great nursing skills have to come in, in terms of communication and just remembering your nonverbals and all of those soft skills that you develop as a nurse over the years. They will never count as much as those moments.” Because a MAID death is rapid, with the patient transitioning from living to death at a pace that often feels startling to families, many RNs made it a practice to prepare family members for what they might see and feel, and what would be happening. As one recalled, “A lot of them just have just straight-on logistical questions. You know, how many medications, what does it look like, you know.” Another said “A few people snore, you know, and kind of have that transition as they are passing away. So, maybe not sitting straight up, maybe having a place where they can lay back or just tilt a little bit.”

In many instances, RNs were present at the time of provision to start the IV, and be witness to the provision. We heard many accounts of their efforts to ‘read the environment’ in order that they could use themselves in a manner that facilitated the best possible experience for all concerned. Some described making themselves ‘invisible’ in the critical moments of provision, so as to optimize the family sense of powerful presence with their dying loved one. Others described taking steps within their communications to speed up or slow down the process, given their sense of what the patient and family needed in that moment. Beyond words, their communications often involved touch in these moments, searching out where reassurance or support might be most needed, and working out how best to provide support so that the memories of the moment were as positive as possible. “I still say, you know, ‘Take care, rest in peace’, to people who've just passed away and I still let them know I'm going to lift up their arm when I move them because that's just the kind of nurse I am.” Finally, recognizing that a MAID death will have changed that family forever, many RNs sought opportunities to engage in the aftermath, ensuring that loved ones were not left dangling with unanswered questions or a sense of a system abandoning them. As one recalled, “You call the funeral home, but it's no big rush.”

It is important to emphasize that these new and evolving communications challenges occurred in the context of what RNs already found to be complex relational care for all patients and families nearing the end of life. As one recalled, “One wife told me a couple weeks ago that, even though she's suffering every single day, having someone in recognizing that she's suffering is all she needs.”

## Discussion

The communicative care that RNs are providing for patients seeking and accessing MAID is highly complex, individualized, and context-specific (Hébert & Asri, 2022). Increasingly, RNs across a range of settings that are not typically understood as death and dying specialty practices are finding themselves engaged with patients who have chosen to seek MAID toward life's end. Therefore, in order to ensure that these patients and their families are well served by the care system during these most vulnerable times, RNs are having to work out how to engage in thoughtful, frank and open conversations about death and dying, and end-of-life options (Variath et al., 2020). The accounts of these RNs illuminate how important this work is and how dedicated they have been to trying to get it right.

While practice standards and best-practice guidelines are helpful in delineating some specific aspects of communicative care, they tend to be somewhat vague with respect to the nuances and intricacies of the everyday communications between patients and RNs (McCabe & Timmins, 2013; Öhlén & Friberg, 2023). In the early days of MAID practice in Canada, RNs were often overwhelmed with the enormous impact of their engagement with patients, and often felt they were operating without clear direction (Beuthin et al., 2018). Over time, they have come to appreciate the complexity associated with integrating what can generally be recommended by way of communication (such as probing questions to open up discussions about goals of care (Sanders et al., 2018), and what must be decided in this particular moment with this particular patient. By engaging communities of care – debriefing cases, holding practice dialogue sessions, seeking continuing education, and so on – they are beginning to feel more comfortable about the boundaries associated with what constitutes communication care excellence and where the potential violations or errors might lie (Frolic et al., 2022). They are increasingly expecting to be well informed about the legislation, practice standards for all members of the team, and the available options within their facilities for optimizing their capacity to meet the patient and family on this issue, and care for them throughout this journey.

In seeking to understand how individual RNs have experienced enacting safeguards and fulfilling their responsibilities within patient communication with respect to MAID, it seems apparent that the implications of their communications with patients will continue to evolve as the eligibility landscape expands. While, in hindsight, the initial experiences for many RNs were relatively straightforward in that the patients having MAID were already on a trajectory toward death, the newer legislation making MAID available to those whose irremediable suffering is due to a condition of a more chronic and ongoing nature means that many will have to be helping patients navigate much less certain terrain, manage increasing levels of expectation, and participate in even more complex conversations (Pesut et al., 2024). This change also means that many RNs not yet exposed to MAID care in their practice will have to rise to the occasion when patients pose questions, seek help in obtaining information, and begin to plan toward assessment and provision. It further means that health profession regulators may need to develop supports and resources for all health care professionals that are increasingly clear on the implications of legislation and practice decisions with respect to end-of-life in general and MAID in particular.

RNs are familiar with the idea of non-standardized conversations, in that communication is very much part of the relational context of their practice across settings and context (Kerr et al., 2022). Their orientation is toward understanding and meeting the unique needs and circumstances of each patient and family (Feldthusen et al., 2022; Kwame & Petrucka, 2021). Among the overarching values shaping nurse patient communication are knowing and respecting patient preference, supportive presence regardless of difficult circumstances, and doing all they can to facilitate a good death experience for all concerned (De Brasi et al., 2021). In that context, RN's accounts are replete with instances of what seem quite diverse communicative acts that they feel have been constructive within a specific and particular patient context, such as using direct or indirect terminology, showing emotions or trying to mask them, engaging in teasing or humour where they have determined it will be well received and appropriate, probing gently or even more firmly as they see the necessity, supporting patient choice without question or encouraging patients to think toward specific directions. Thus, ‘getting it right’ for RNs is highly context dependent, significantly informed by their capacity to establish and sustain good relational practice, and on their skill with respect to ‘knowing’ the patient. While there is a tendency within the literature pertaining to complex practices such as MAID communication to try to articulate generalized knowledge and recommendations (Oczkowski et al., 2021), many RNs realize that moving beyond routine practice and toward person- or patient-centred care in this regard will require ongoing assessments of what patients are trying to communicate and what the RN needs to know in order to individualize their specific experiences.

It is important also to acknowledge that this complex communication practice is also being conducted concurrently with the ongoing moral wrestling that many RNs engage with as they reflect on MAID practice in general, the evolving conditions under which patients may become eligible for MAID, and the specific knowledge they may have about the individual circumstances in which each individual patient may be requesting it (Dorman & Raffin Bouchal, 2020). Because they generally do not have direct input into the eligibility assessment, RNs may feel that the basis for that decision reflects partial or biased knowledge, rather than the more fulsome understanding of that patient's circumstances that they may have formed over time. In addition, they may well have been actively supporting these patients in their suffering, feel personally responsible if it has not been resolved in a manner they believe theoretically possible, and therefore may be holding a complex moral burden with respect to their own values and beliefs while also supporting eligible patients and their families toward the best death possible.

### Strengths & limitations

We are aware that our sampling for this study was by convenience – we interviewed any RNs who self-identified as having some involvement with MAID and were willing to speak with us about it. Our sample may therefore have included more RNs with a favorable attitude toward it, and therefore who may have put more thought into how best to support patients going through it than would some of their colleagues more focused on avoiding exposure to the practice. It is also certainly possible that those who chose to reveal the complexities of their practices, including those about which they felt a bit uncertain, might be those most familiar with the legislation and its implications. RNs who may have an equal degree of engagement with patients seeking MAID but who are unfamiliar with practice expectations may have preferred not to expose themselves to a challenge.

Nevertheless, a strength of this study is the number of RNs from a diversity of practice contexts who openly and frankly shared their thoughts and feelings about this phenomenon, in addition to recounting instances from their clinical practice that were illustrative of the care complexities they faced. The richness and depth of their accounts, and the willingness of so many RNs to allow us into their philosophical, moral and ethical wranglings provided us with a solid foundation of understanding on which to begin to support the processes of ongoing study, practice guidelines, and clinical supports. This has also allowed us a strong basis for arguing for the active engagement in more RN voices within the national and regional MAID planning and policy processes, as it is very clear that these nurses often have powerfully influential roles in the total package of MAID care.

### Implications

Although we think that seeking standardized communication practice guidelines for MAID-related care may be misguided, the insights of these RNs make it quite clear that an enhanced understanding of what might constitute poor practice will be essential. As more and more RNs encounter MAID in the context of their practice, the profession must be on guard for strategies to protect patients from the potential harms that could be associated with nursing communication (such as bias, moralizing, or judgmental attitudes), and must continue to try to articulate practice ideals that are consistent with the vision of human dignity, autonomy and individual choice that the MAID legislation represents. Educational content in this regard will have to be integrated into both basic and continuing education for RNs across a wide range of practice settings, and clinical setting leaders will need to ensure that there are clearly articulated supports available to nurses as their practice expands in this respect. Further, ongoing research will be required across the spectrum of RN practice contexts as new dimensions of the MAID communication challenge come to light and conditions change over time.

## Conclusion

The findings of this study help convey the high degree of complexity, nuance and challenge involved in the communicative work of RNs with patients in the process of seeking or receiving MAID in Canada. These challenges often reflect new practice dimensions that were never or rarely encountered prior to the advent of MAID in 2016 and are also evolving over time such that what was true even a year or two ago may no longer be the case today. Many of these are RNs who have not specifically sought out a career in the field of death and dying, and who have been brought into rapidly learning about the practice by virtue of patients under their care who choose this option. They will not always have had any formal educational opportunities with respect to the implications of MAID care, and the available evidence to inform best practices in this regard is still in its infancy.

It is therefore remarkable to learn from these reports of their experiences with MAID how sophisticated and complex RN thinking about the phenomenon has become in a relatively short period of time. As researchers, we have been in awe of the creative, imaginative, and deeply human ways in which RNs are learning to support the processes through which MAID is being made available to Canadians who are taking this legally available option to exert autonomy over the timing, manner and form of their own death.

## References

[bibr1-08445621241281993] Acorn M. Byres D. (2020). The integration of advance practice nursing roles in Canada. In Staples E. Pilon R. Hannon R. A. (Eds.), Canadian Perspectives on advanced practice nursing (2nd ed., pp. 50–60). Canadian Scholars.

[bibr2-08445621241281993] Banner D. Schiller C. J. Freeman S. (2019). Medical assistance in dying: A political issue for nurses and nursing in Canada. Nursing Philosophy, 20(4), e12281. 10.1111/nup.12281 31478340

[bibr3-08445621241281993] Beuthin R. Bruce A. Scaia M. (2018). Medical assistance in dying (MAiD): Canadian nurses’ experiences. Nursing Forum, 53(4), 511–520. 10.1111/nuf.12280 29972596 PMC6282783

[bibr4-08445621241281993] Canadian Association of MAID Assessors and Providers [CAMAP]. (2022). Canadian MAID curriculum. https://camapcanada.ca/curriculum/

[bibr5-08445621241281993] Canadian Nurses Protective Society. (2021, July). Medical Assistance in Dying: What every nurse should know. https://cnps.ca/article/medical-assistance-in-dying-what-every-nurse-should-know/#:∼:text=In%20fact%2C%20the%20Criminal%20Code,about%20MAID%20with%20their%20patients

[bibr6-08445621241281993] Close E. Jeanneret R. Downie J Willmott L. White B.P. (2023). A qualitative study of experiences of institutional objection to medical assistance in dying in Canada: Ongoing challenges and catalysts for change. BMC Medical Ethics 24(1), 71. 10.1186/s12910-023-00950-9 37735387 PMC10512474

[bibr7-08445621241281993] De Brasi E. L. Giannetta N. Ercolani S. Gandini E. L. M. Moranda D. Villa G. Manara D. F. (2021). Nurses’ moral distress in end-of-life care: A qualitative study. Nursing Ethics, 28(5), 614–627. 10.1177/0969733020964859 33267730

[bibr8-08445621241281993] Dorman J. D. Raffin Bouchal S. (2020). Moral distress and moral uncertainty in medical assistance in dying: A simultaneous evolutionary concept analysis. Nursing Forum, 55(3), 320–330. 10.1111/nuf.12431 31957042

[bibr9-08445621241281993] Downar J. Fowler R. A. Halko R. Huyer L. D. Hill A. D. Gibson J. L. (2020). Early experience with medical assistance in dying in Ontario, Canada: A cohort study. CMAJ: Canadian Medical Association Journal = Journal de L'Association Medicale Canadienne, 192(8), E173–E181. 10.1503/cmaj.200016 PMC7043822 32051130

[bibr10-08445621241281993] Downar J. MacDonald S. Buchman S. (2023). Medical assistance in dying and palliative care: Shared trajectories. Journal of Palliative Medicine, 26(7), 896–899. 10.1089/jpm.2023.0209 37428971

[bibr11-08445621241281993] Dumsday T. (2023). From a court judgement to federal law. In Kotalik J. Shannon D. W. (Eds.), Medical assistance in dying (MAID) in Canada: Key multidisciplinary perspectives (pp. 55–67). Springer. 10.1007/978-3-031-30002-8_3

[bibr12-08445621241281993] Feldthusen C. Forsgren E. Wallström S. Andersson V. Löfqvist N. Sawatzky R. Öhlén J. Ung E. J. (2022). Centredness in health care: A systematic overview of reviews. Health Expectations, 25(3), 885–901. 10.1111/hex.13461 35261138 PMC9122448

[bibr13-08445621241281993] Frolic A. Miller P. Harper W. Oliphant A. (2022). MAid to last: Creating a care ecology for sustainable medical assistance in dying services. HEC Forum: An Interdisciplinary Journal on Hospitals’ Ethical and Legal Issues, 34(4), 409–428. 10.1007/s10730-022-09487-7 36094775 PMC9465134

[bibr14-08445621241281993] Government of Canada (2016, June 17). Criminal Code of Canada section 241.2 from 2016-06-17 to 2021-03-16. https://laws-lois.justice.gc.ca/eng/acts/C-46/section-241.2-20160617.html

[bibr15-08445621241281993] Government of Canada. (2021, March 16). Criminal Code of Canada section 241.2 from 2021-03-16. https://laws-lois.justice.gc.ca/eng/acts/C-46/section-241.2.html

[bibr16-08445621241281993] Government of Canada. (2024, April 25). Criminal Code of Canada (R.S.C., 1985, c. C-46): Counseling or aiding suicide. https://laws-lois.justice.gc.ca/eng/acts/c-46/section-241.html

[bibr17-08445621241281993] Health Canada. (2023a, June 30). Advice to the profession: Medical Assistance in Dying (MAID). https://www.canada.ca/en/health-canada/services/publications/health-system-services/advice-profession-medical-assistance-dying.html#a19

[bibr18-08445621241281993] Health Canada. (2023b, October). Fourth annual report on Medical Assistance in Dying in Canada 2022. https://www.canada.ca/en/health-canada/services/publications/health-system-services/annual-report-medical-assistance-dying-2022.html

[bibr19-08445621241281993] Health Canada. (2024a, February 1). Government of Canada introduces legislation to delay medical assistance in dying expansion by 3 years. https://www.canada.ca/en/health-canada/news/2024/02/the-government-of-canada-introduces-legislation-to-delay-medical-assistance-in-dying-expansion-by-3-years.html#:∼:text=The%20Government%20of%20Canada%20introduces%20legislation%20to%20delay%20Medical%20Assistance%20in%20Dying%20expansion%20by%203%20years

[bibr20-08445621241281993] Health Canada. (2024b, March 5). Medical assistance in dying: Overview. https://www.canada.ca/en/health-canada/services/health-services-benefits/medical-assistance-dying.html

[bibr21-08445621241281993] Hébert M. Asri M. (2022). Paradoxes, nurses’ roles and medical assistance in dying: A grounded theory. Nursing Ethics, 29(7-8), 1634–1646. 10.1177/09697330221109941 35758866 PMC9667088

[bibr22-08445621241281993] Joolaee S. Ho A. Serota K. Hubert M. Buchman D. Z. (2022). Medical assistance in dying legislation: Hospice palliative care providers’ perspectives. Nursing Ethics, 29(1), 231–244. 10.1177/09697330211012049 34538192 PMC8866752

[bibr23-08445621241281993] Kerr D. Martin P. Furber L. Winterburn S. Milnes S. Nielsen A. Strachan P. (2022). Communication skills training for nurses: Is it time for a standardised nursing model? Patient Education and Counseling, 105(7), 1970–1975. 10.1016/j.pec.2022.03.008 35301988

[bibr24-08445621241281993] Kwame A. Petrucka P. M. (2021). A literature-based study of patient-centered care and communication in nurse-patient interactions: Barriers, facilitators, and the way forward. BMC Nursing, 20(1), 158. 10.1186/s12912-021-00684-2 34479560 PMC8414690

[bibr25-08445621241281993] McCabe C. Timmins F. (2013). Communication skills for nursing practice. Bloomsbury Publishing.

[bibr26-08445621241281993] Oczkowski S. J. W. Crawshaw D. Austin P. Versluis D. Kalles-Chan G. Kekewich M. Curran D. Miller P. Q. Kelly M. Wiebe E. Dees M. Frolic A. (2021). How we can improve the quality of care for patients requesting Medical Assistance in Dying: A qualitative study of health care providers. Journal of Pain and Symptom Management, 61(3), 513–521. 10.1016/j.jpainsymman.2020.08.018 32835830

[bibr27-08445621241281993] Öhlén J. Friberg F. (2023). Person-centred conversations in nursing and health: A theoretical analysis based on perspectives on communication. Nursing Philosophy, e12432. Advance online publication. 10.1111/nup.12432 36973865

[bibr28-08445621241281993] Parker H.L. (2024). Nurses’ communicative practices related to medical assistance in dying (MAiD). [Unpublished master’s thesis, University of British Columbia - Okanagan]. https://open.library.ubc.ca/soa/cIRcle/collections/ubctheses/24/items/1.0444133?o=5

[bibr29-08445621241281993] Pesut B. Thorne S. Chambaere K. Hall M. Schiller C. (2024). The evolving complexities of MAID care in Canada from a nursing perspective. Global Qualitative Nursing Research, 11, 23333936241228233. 10.1177/23333936241228233 38433773 PMC10908223

[bibr30-08445621241281993] Pesut B. Thorne S. Greig M. (2020a). Shades of gray:” conscientious objection in medical assistance in dying. Nursing Inquiry, 27(1), e12308. https://onlinelibrary.wiley.com/doi/full/10.1111/nin.12308 https://doi.org/10.1111/nin.12308 10.1111/nin.12308 PMC7027545 31273903

[bibr31-08445621241281993] Pesut B. Thorne S. Greig M. Chambaere K. Storch J. Burgess M. (2020b). Riding an elephant: Understanding nurses’ moral journeys in the context of MAiD. Journal of Clinical Nursing, 29(19-20), 3870–3881. 10.1111/jocn.15427 32700402 PMC7540490

[bibr32-08445621241281993] Pesut B. Thorne S. Greig M. Chambaere K. Tishelman C. Roussel J. (2020c). Constructing “good nursing practice” for medical assistance in dying: A qualitative interpretive study. Global Qualitative Nursing Research, 7, 2333393620938686. https://www.ncbi.nlm.nih.gov/pmc/articles/PMC7377599/ https://doi.org/10.1177/2333393620938686 32743024 10.1177/2333393620938686 PMC7377599

[bibr33-08445621241281993] Pesut B. Thorne S. Greig M. Schiller C. Roussel J. (2020d). The rocks and hard places of MAiD: A qualitative study of nursing practice in the context of legislated assisted death*.* BMC Nursing, 19, Art. 12. https://bmcnurs.biomedcentral.com/track/pdf/10.1186/s12912-020-0404-5 https://doi.org/10.1186/s12912-020-0404-5 10.1186/s12912-020-0404-5 PMC7025406 32095114

[bibr34-08445621241281993] Pesut B. Thorne S. Purveen G. Leimbigler B. (2023). Supporting nursing roles in Medical Assistance in Dying: Development and evaluation of an evidence-based reflective guide. PEC Innovation, 3, 100234. 10.1016/j.pecinn.2023.100234 38090105 PMC10711158

[bibr35-08445621241281993] Pesut B. Thorne S. Stager M. L. Schiller C. Penny C. Hoffman C. (2019). Medical assistance in dying: A narrative review of Canadian nursing regulatory documents. Policy, Politics, & Nursing Practice, 20(3), 113–130. 10.1177/1527154419845407 PMC6827351 31060478

[bibr36-08445621241281993] Pesut B. Wright D. K. Thorne S. Hall M. Purveen G. Storch J. Huggins M. (2021). What’s suffering got to do with it? A qualitative study of suffering in the context of medical assistant in dying (MAiD). BMC Palliative Care, 20(1), Art. 174. 10.1186/s12904-021-00869-1 PMC8582137 34758799

[bibr37-08445621241281993] Sanders J. J. Curtis J. R. Tulsky J. A. (2018). Achieving goal-concordant care: A conceptual model and approach to measuring serious illness communication and its impact. Journal of Palliative Medicine, 21(S2), S17–S27. 10.1089/jpm.2017.0459 PMC5756461 29091522

[bibr38-08445621241281993] Sawatzky R. Porterfield P. Lee J. Dixon D. Lounsbury K. Pesut B. Roberts D. Tayler C. Voth J. Stajduhar K. (2016). Conceptual foundations of a palliative approach: A knowledge synthesis. BMC Palliative Care, 15, 5. 10.1186/s12904-016-0076-9 26772180 PMC4715271

[bibr39-08445621241281993] Thorne S. (2013). Secondary qualitative data analysis. In Beck C. T. (Ed.), Routledge international handbook of qualitative nursing research (pp. 393–404). Taylor & Francis.

[bibr40-08445621241281993] Thorne S. (2016). Interpretive description: Qualitative research for applied practice (2^nd^ ed.). Routledge.

[bibr41-08445621241281993] Variath C. Peter E. Cranley L. Godkin D. Just D. (2020). Relational influences on experiences with assisted dying: A scoping review. Nursing Ethics, 27(7), 1501–1516. 10.1177/0969733020921493 32436431

[bibr42-08445621241281993] Wiebe E. Green S. Wiebe K. (2021). Medical assistance in dying (MAiD) in Canada: Practical aspects for healthcare teams. Annals of Palliative Medicine, 10(3), 3586–3593. 10.21037/apm-19-631 32787380

[bibr43-08445621241281993] Winters J. P. Pickering N. Jaye C. (2021). Because it was new: Unexpected experiences of physician providers during Canada's early years of legal medical assistance in dying. Health Policy (Amsterdam, Netherlands), 125(11), 1489–1497. 10.1016/j.healthpol.2021.09.012 34629201

